# Animal Models of Chronic Hepatitis Delta Virus Infection Host–Virus Immunologic Interactions

**DOI:** 10.3390/pathogens4010046

**Published:** 2015-02-12

**Authors:** Rafael Aldabe, Lester Suárez-Amarán, Carla Usai, Gloria González-Aseguinolaza

**Affiliations:** Gene Therapy and Regulation of Gene Expression Program, Centro de Investigación Médica Aplicada (CIMA), Universidad de Navarra (UNAV), Pamplona 31008, Spain; E-Mails: raldabe@unav.es (R.A.); lsuarez.4@alumni.unav.es (L.S.-A.); cusai@alumni.unav.es (C.U.)

**Keywords:** hepatitis delta virus, HDV animal models, liver damage, antiviral treatment, vaccines

## Abstract

Hepatitis delta virus (HDV) is a defective RNA virus that has an absolute requirement for a virus belonging to the hepadnaviridae family like hepatitis B virus (HBV) for its replication and formation of new virions. HDV infection is usually associated with a worsening of HBV-induced liver pathogenesis, which leads to more frequent cirrhosis, increased risk of hepatocellular carcinoma (HCC), and fulminant hepatitis. Importantly, no selective therapies are available for HDV infection. The mainstay of treatment for HDV infection is pegylated interferon alpha; however, response rates to this therapy are poor. A better knowledge of HDV–host cell interaction will help with the identification of novel therapeutic targets, which are urgently needed. Animal models like hepadnavirus-infected chimpanzees or the eastern woodchuck have been of great value for the characterization of HDV chronic infection. Recently, more practical animal models in which to perform a deeper study of host virus interactions and to evaluate new therapeutic strategies have been developed. Therefore, the main focus of this review is to discuss the current knowledge about HDV host interactions obtained from cell culture and animal models.

## 1. Introduction

### 1.1. The Disease, Chronic HDV Infection

Worldwide there are approximately 350 million individuals chronically infected with the hepatitis B virus (HBV); among them 15 to 20 million are coinfected with hepatitis delta virus (HDV) [1]. HDV was first described in 1977 when a novel antigen was detected in the nucleus of hepatocytes from patients chronically infected with HBV who developed serious episodes of severe liver disease [2]. In 1980 the same research group discovered that this nuclear antigen was derived from a new virus that they named hepatitis delta virus [3]. Thus, HDV was established as the causative infectious agent responsible for exacerbation of liver disease in HBV-infected patients [4,5]. HBV/HDV coinfection is associated with more severe acute hepatitis, higher risk of cirrhosis and decompensated liver disease, and higher mortality than HBV monoinfection. From 1990, after the initiation of the universal HBV vaccination campaigns, the incidence of HBV and concomitantly of HDV declined in the developed world. However, during the last 10 years, epidemiological studies revealed a stabilization, and in some cases an increase, in the incidence of HDV in several countries [6,7,8,9,10,11]. Furthermore, the epidemiology of HDV has not changed in developing countries where the HBV vaccine is not being administered to the population. Thus, HDV chronic infection represents a major health problem worldwide. Unfortunately, the treatment options for chronic HDV are limited. The only drug in use is pegylated interferon-α (PEG-IFN-α), which is associated with a low rate of cure and a significant number of patients in whom the virus relapses after cessation of treatment [12,13,14]. In addition, PEG-IFN-α is associated with a variety of adverse events, including flu-like symptoms, neuropsychiatric events, anemia, and thrombocytopenia. Thus, more effective treatment options are needed. The particular characteristics of HDV make the development of new therapeutic approaches very difficult. HDV depends on the activity of cellular enzymes to complete its life cycle and does not code for viral enzymes, which in the case of other viruses represent the main targets for the development of specific antivirals.

New treatments based on the use of interference RNA molecules [15,16] or—peptides to inhibit HDV-HBsAg interaction to block viral entry into cells—are being explored [17,18,19,20,21]. Moreover, inhibition of enzymatic processes that are required by the virus to complete its life cycle, e.g. the prenylation of viral antigens, represents a very interesting target [22,23]. Currently the antiviral activity of two prenylation inhibitors is being tested by Eiger BioPharmaceuticals in phase 2 clinical trials; these drugs are expected to become the first “specific” treatment for HDV.

### 1.2. Main Characteristics of HDV

**Figure 1:**
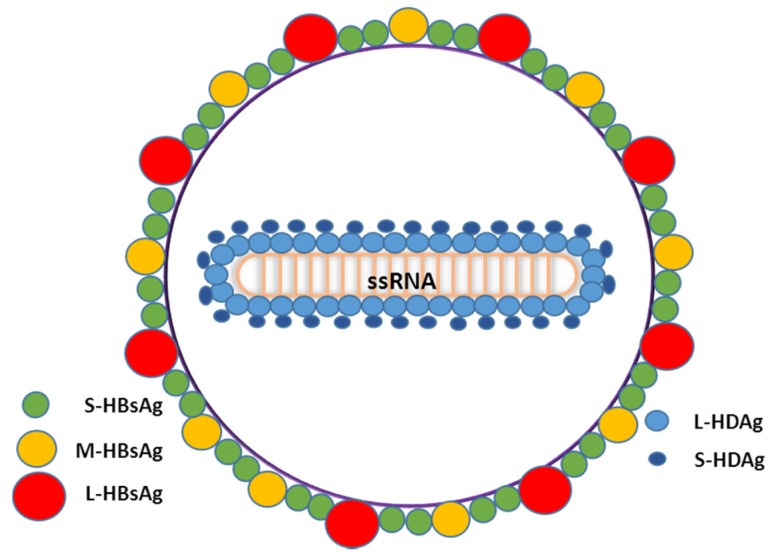
Schematic representation of HDV virions describing all the components of the viral particle. l-HDAg: HDV Large antigen; S-HDAg: HDV short antigen; S-HBsAg: Small HBV surface antigen; M-HBsAg: Medium HBV surface antigen; l-HBsAg: Large HBV surface antigen; ssRNA: single-stranded RNA.

HDV is the only member of the genus Deltavirus. HDV is a defective RNA virus that requires the HBV surface antigens (HBsAg) for viral assembly and transmission [14,24]. HDV virion is a small and spherical hybrid particle with a diameter of about 36–46 nm [24]. It is composed of the HDV RNA genome and about 200 molecules of hepatitis delta antigen (HDAg) enclosed by the hepatitis B surface antigen (HBsAg) and host lipids membrane (Figure 1). The HBsAg envelope protects the viral genome from the extracellular environment and dictates the hepatocyte tropism of HDV.

The HDV genome is a circular negative sense RNA molecule of approximately 1700 nucleotides with the ability to fold on itself, appearing as a double-stranded rod-like structure with approximately 70% intramolecular base-pairing [25,26,27]. Its structure and function is very similar to the genome of plant viroids. In fact, replication of the HDV genome involves a rolling circle mechanism, requiring autocatalytic cleavage of the RNA by an internal ribozyme analogous to that proposed for plant viroids [28,29,30]. HDV genome replication involves only RNA species; in addition to the genome, infected cells contain, in smaller amounts, an exact complementary RNA species, called the antigenome, and much smaller amounts of a messenger RNA with a 5'-cap and a 3'-poly(A) tail. HDV isolates have been divided in eight clades based on nucleotide sequence and geographic distribution. The different isolates can differ in more than 30% of their nucleotide sequence [31].

### 1.3. Virus Life Cycle

**Figure 2:**
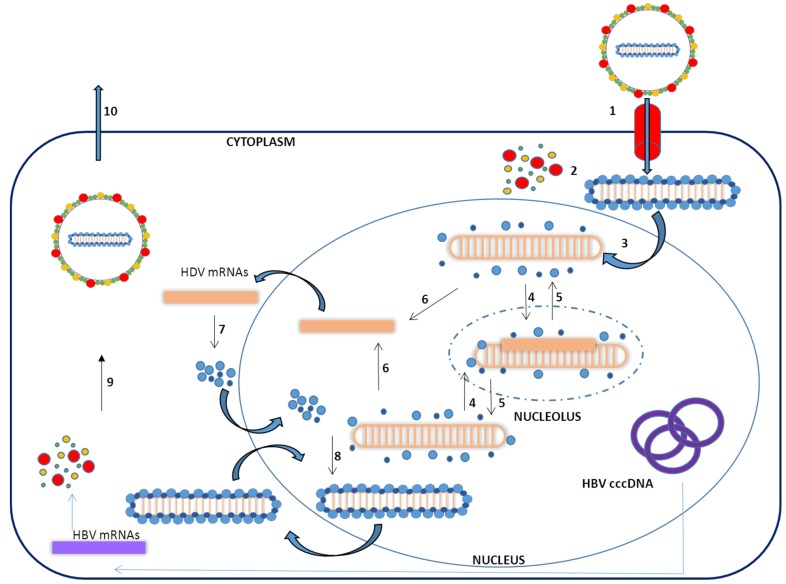
Schematic representation of the HDV viral cycle: (**1**) Binding to NTCP on human hepatocyte; (**2**) Uncoating; (**3**) Translocation of the ribonucleoprotein particle into the nucleus; (**4**) Transcription of the antigenome in the nucleolus.; (**5**) Production of genomic RNA in the nucleoplasm; (**6**) Transcription of the mRNA; (**7**) Translation of HDAg; (**8**) Ribonucleoparticle assembly; (**9**) Asotiation of HBsAg Virion production in the cytoplasm; (**10**) Virion release. HBV cccDNA: HBV covalently closed circular DNA. HBV mRNAs: HBV messenger RNAs.

To complete a cycle of HDV infection the hepatocyte must be infected by both HDV and HBV, since the formation of new viral particles requires HBV surface antigens. HDV infectivity is dependent upon a receptor-binding motif in the N-terminal region of the pre-S1 domain of the HBsAg [18,32]. Very recently, the receptor for both HBV and HDV infection has been identified as the human sodium-taurocholate cotransporting polypeptide (hNTCP) [32,33,34]. The NTCP is a transmembrane transporter present on the basolateral membrane of hepatocytes and is responsible for the uptake of conjugate bile acids from enterohepatic circulation [35]. After entering the hepatocytes, the virus is uncoated and a signal in the HDAg amino acid sequence mediates the traslocation of the nucleocapsid into the nucleus [36]. To replicate its genome, the virus uses the host RNA polymerase/s [37]. Once inside the nucleus, cellular RNA polymerases synthesize the antigenomic RNA in the nucleolus and genomic RNA in the nucleoplasm (Figure 2) [26,37,38,39,40]. There is some controversy about which types of cellular RNA polymerases are involved in HDV RNA replication. Host RNA polymerases I, II, and III have been shown to interact with the HDV genome [41]. However, while RNA polymerase II was consistently found to interact with HDV components; the interaction with RNA polymerases I and III was not that clear [42].

Studies performed by different groups, particularly by Taylor and colleagues, indicated that the replication of the RNA genome, antigenome, and messenger RNA of HDV involves RNA polymerase II [43,44]. This is mainly substantiated by the sensitivity of HDV RNA replication to the RNA polymerase inhibitor α-amanitin. However, the role of RNA polymerases I and III is still under consideration. RNA polymerase inhibition studies demonstrated *de novo* synthesis of HDV antigenome RNA molecules in the presence of a RNA polymerase II inhibitor, while its synthesis is decreased in the presence of RNA polymerase I inhibitor, suggesting that RNA polymerase I might be involved in this process [45]. More studies need to be performed to clarify these issues; however, what is clear is that the enzyme mainly involved in this HDV replication is RNA polymerase II.

HDV RNA is synthesized first as linear concatameric RNA that contains many copies of the genome. [28,30]. Then, the 85-nt site-specifc viral ribozyme, found on each RNA strand, acts to process the RNA transcripts to unit lengths, monomers. These monomers are then ligated to form circular RNA. It is unclear if ligation is carried out by the ribozyme or a host RNA ligase [46]. During HDV replication, it is possible to detect three forms of HDV RNA: circular genomic RNA, circular complementary antigenomic RNA, and a linear polyadenylated antigenomic RNA, which is the mRNA template containing the open reading frame for the translation of the hepatitis delta antigen (HDAg) [47]. There are two isoforms of the HDAg: the 24 kDa small form (S-HDAg), with 195 amino acid residues, and the 27 kDa large form (l-HDAg) with 214 amino acids [47]. The amino acid sequence of both isoforms is identical in the N-terminus, but l-HDAg is 19 amino acids longer at the C-terminus than S-HDAg.

S-HDAg is encoded by the viral genome and l-HDAg is the result of an RNA editing event mediated by host enzyme adenosine deaminase-1 (ADAR-1). ADAR-1 replaces the stop codon UAG at position 196 with a tryptophan codon UGG, extending the open reading frame and leading to the production of the large antigen [48,49,50,51]. The 19 extra amino acids added at the carboxyl terminal end of l-HDAg confer its functional properties that are different from S-HDAg. S-HDAg is required for the initiation of the viral genome replication; it is essential for the transcription and accumulation of newly processed HDV RNAs, whereas l-HDAg, which is synthesized in the late stage of viral replication, serves as a principal inhibitor of replication and is essential in the assembly of HDV virions. l-HDAg, by inhibiting viral replication, also regulates its own synthesis since it prevents the editing of the amber/W site necessary for the expression of l-HDAg. Furthermore, the HDV genome and antigenome associate with multiple copies of the S-HDAg and l-HDAg, forming ribonucleoprotein complexes (RNPs). Only the RNPs containing the HDV genomic molecule interact with HBV surface molecules and form new viral particles [52].

Both antigens suffer posttranslational modifications, which are very important to modulate their functions. S-HDAg is acetylated, phosphorylated, and sumoylated and l-HDAg is acetylated, phosphorylated, and isoprenylated. S-HDAg phosphorylation is crucial for its interaction with cellular RNA polymerase II and RNA binding. Sumoylation of S-HDAg results in an increase in HDV genomic RNA and mRNA synthesis. Acetylation of both antigens modulates HDV replication. Acetylation and phosphorylation of l-HDAgs are essential for the correct trafficking of this antigen inside the nucleus (nucleolus-nuclear speckles) and secretion. Isoprenylation of the l-HDAg is essential for the interaction of l-HDAg with HBSAg and the formation of new virions. The exact location where l-HDAg and HBsAg antigens interact is not well known; isoprenylated l-HDAg is mainly located in the nucleus and HBsAg is in the cytoplasm, thus l-HDAg forming part of HDV RNPs needs to be exported from the nucleus to the cytoplasm. The elucidation of the mechanism involved in this step requires more studies.

HDV viral particle generation depends on HBV surface antigen production; however, it is still not clear if HDV infection can persist in the absence of HBV replication. Contradictory data about this issue are available in the literature. It has been reported that HDV RNA, when introduced in human cells in the absence of HBV, can replicate efficiently, produce HDAgs, and form RNPs that are not able to exit the cell. Furthermore, if another alternative source of HBV envelope proteins is available, like integrated HBV DNA, HDV can replicate itself independently of hepadnavirus replication [53]. In fact, HCC cells that apparently no longer support HBV replication but can still produce the envelope proteins from HBV integrants can generate HDV infectious particles [54]. Recent studies performed in mice with humanized livers showed that HDV monoinfection can persist on human hepatocytes for at least 6 weeks and can be rescued by HBV superinfection [55]. In addition, data in a very small number of patients showed that HDV can survive and synthesize HDAg in the absence of detectable HBV [56], However, on the other hand, it has been demonstrated that inhibition of hepadnavirus infection in patients and animal models by the use of antiviral medication led to reduction or loss of HDV replication [57,58]. Thus, nowadays the consensus is that HDV can persist without HBV but only for a brief period of time.

## 2. *In Vitro* HDV–Host Cell Interaction

Although a wide range of cultured cell lines can support HDV replication (HEK-293, HeLa, Huh7) after the transfection of a plasmid containing the HDV genome, the virus fails to infect any cultured cell lines except primary cultures of hepatocytes and Hepa RG cells with a very low efficiency [59]. However, very recently this situation has dramatically changed with the discovery of the HBV/HDV receptor. Human as well as other species cell lines that stably express the sodium taurocholate receptor have been generated; these cells are infected by and are able to sustain HDV replication [32,33,34].

In order to elucidate the mechanism responsible for the increase in liver pathology observed in patients infected with HDV, several authors have analyzed the interaction of viral components with cell components* in vitro* or in cell culture. The first experiments performed to identify host factors interacting with HDAg were performed using a yeast two-hybrid system [60,61]. From these studies a single protein was identified and named as delta antigen interacting protein A or DIPA. DIPA is a cellular protein with high homology to HDAg. DIPA overexpression inhibits HDV replication; however, confirmatory studies and a deeper analysis about the mechanism are lacking. Later on, different proteomic approaches have been performed in a variety of cell lines, identifying several factors that interact with delta antigen such as subunits of RNA polymerase II complex, hnRNP proteins, RNA helicases, the histone H1, PML, nucleolin, B23, Pol I specific factor SL1, the negative transcription elongation factor DSIF, and PKR [42]—all of these factors have been suggested to affect HDV replication, but only in some cases experimentally proven.

Furthermore, it has been shown that the HDV RNAs interact with the polypyrimide tract-binding protein-associated splicing factor (PSF), PKR, GAPDH, and ADAR-1 [62,63,64,65]. In fact, as previously indicated, ADAR-1 has been shown to be responsible for HDV antigenome editing [51].

Regarding the potential mechanism of HDV-mediated cytotoxicity, it has been reported that cells expressing HDV proteins present multiple cellular signaling pathways modified like enhanced ROS production, leading to oxidative stress and activation of several transcription factors such as STAT-3 or NF-κB [66] or potentiated TGF-β signal activation [67]. These HDV-mediated cellular modifications can promote several disease phenotypes associated with HDV infection like liver fibrosis or cellular damage. Moreover, HDV can interfere with the JAK-STAT signal transduction pathway in response to IFN-α, impairing the phosphorylation of both STAT1 and STAT2 and leading to inefficient IFN-α stimulated gene expression [68], which might explain the relatively low antiviral efficacy of PEG-IFN-α.

There is also evidence suggesting that both HDAgs are transcriptional inducers, as both are capable of regulating cellular gene expression presenting l-HDAg broader transcriptional activity [69,70,71]. One mechanism mediating such effects can be histone acetylation, as an upregulation of this modification associated with expression of both HDAgs and HDV replication has been observed [72].

Proteomic analysis of cells producing whole HDV RNPs shows a set of changes in host protein synthesis that in some cases seems to correlate with previous pathologic findings observed in both chronic and acute HDV patients, like changes in enzymes involved in lipid metabolism [63]. Furthermore, this study indicates an overall deregulation of DNA replication and cell cycle control in liver cells, like downregulation of PCNA and the Fen 1 endonuclease, proteins involved in DNA replication. This observation is in accordance with the subtle growth disadvantage associated with HDV replication in dividing cells and deregulation of cell cycle control [73,74]. Regarding innate immune responses, there are no data in the literature describing molecules implicated in the detection of the virus or pattern recognition receptors that detect HDV. However, it has been shown that elements of the virus are able to transactivate the IFN-α-inducible MxA gene [66] and to activate PKR [75].

## 3. Chronic HDV Infection Animal Models

### 3.1. “Natural” Animal Models

In a natural setting, only humans are known to acquire HDV infection [24]; however, it is quite possible that chimpanzees or other large primates can be infected with HDV in the wild. As previously described, the HDV virus requires help from a virus of the family hepadnaviridae for formation of new virions, thus the host range of HDV is limited to those species that support the replication of HBV, like chimpanzees, or a hepadnavirus capable of supplying helper functions [76,77,78,79,80]. Among members of the Hepadnaviridae family, the closest to HBV are the woolly monkey hepatitis B virus (WMHBV) and the woodchuck hepatitis virus (WHV), which can assist in HDV propagation because both encode viral envelope proteins that are competent for HDV ribonucleoprotein (RNP) envelopment [81]. In contrast, the envelope protein of duck hepatitis B virus (DHBV), the most distantly related hepadnavirus, is unable to assist in HDV propagation due to the inability of the envelope protein to package HDV RNP [82]. Recently, bats have been described as a new animal model that could support HDV replication based on the presence of a HBV antigenically related pathogenic hepadnavirus that can generate HDV pseudotyped particles able to infect human and bat primary hepatocytes [83]. Based on its close phylogenetic relation to primates, the tree shrew species *Tupaia belangeri* has been used for infection studies both with HBV and WMHBV [21,84,85], and is also a host for HDV infection [86].

Among these models, the most frequently used for experimental studies is the woodchuck chronically infected with WHV and superinfected with HDV, obtaining interesting and relevant data. Experimental transmission of HDV has been accomplished in the chimpanzee and the eastern woodchuck, hosts of HBV and WHV, respectively [76,87]. In both models upon HDV infection, there is a major suppression of HBV and WHV replication at the peak of HDV replication, as has been observed in humans [88]. Eighty to one hundred percent of HDV superinfections in WHV carrier woodchucks resulted in chronic HDV infection, while coinfection resulted in an acute self-limited infection.

It has been shown that woodchucks treated with cyclosporine A, as an inhibitor of the immune response, showed an increase in HDV viremia and at the same time a reduction of WHV viral load. These results indicate that the host immune response exerts a negative control over HDV replication and that HDV influences HBV replication independently of the host immune response [89]. Furthermore, this model was used during the last years to test vaccination strategies designed to protect HBV carriers from HDV superinfection [90,91,92,93,94].

These natural animal models have provided very useful data and they have allowed for determining the main characteristics of the HDV infective cycle and some immunological responses as they recapitulate several of the biological phenomena observed in humans. However, there are several limitations for performing experiments in these animal models that have delayed the HDV research field, ranging from the relatively large size and genetic variability of these animals to availability and ethical considerations. Furthermore, several issues limit the number of animals that can be used, affecting the statistical significance of any potential results. On top of that, the experimental tools available to work with most of these animal species are very limited, highlighting the need for the development of more practical animal models.

### 3.2. Mouse Models of HDV Replication

#### 3.2.1. Injection of Mice with HDV Infectious Particles

The mouse represents the premier mammalian model system for disease research. Scientists from a wide range of biomedical fields have gravitated to the mouse because of its close genetic and physiological similarities to humans, as well as the ease with which its genome can be manipulated and analyzed. Additionally, scientists have developed a broad range of technological tools and approaches to dissect molecular and physiological processes in one organism that is easy to maintain. Therefore, there have been several attempts by scientists aimed at developing mice as an HDV experimental system.

The first proof of concept study of HDV infection in mice was performed in 1993, by the group of Dr. Taylor at the Fox Chase Cancer Center in Philadelphia; they showed that human hepatitis delta virus (HDV) obtained from the serum of an experimentally infected woodchuck and injected into either the peritoneal cavity or the tail vein of mice was able to infect the murine liver. In this model, genomic HDV increased in the liver during the first 5 to 10 days postinoculation; antigenomic RNA was also detected, and delta antigen was present in the nuclei of the hepatocytes [44]. The infection was liver-specific but involved no more than 0.6% of the mouse hepatocytes. Once HDV entered mouse hepatocytes, the levels of HDV genome replication were comparable to those obtained in a natural infection of woodchuck hepatocytes; however, the infection was short lived, with a half-life of clearance of about 3 days. Moreover, no obvious cytopathic effects were detected.

The reason for the disappearance of the viral genome from the liver was not determined, but the role of T- and B-cell-dependent immune response can be excluded, since the rate of this decrease was the same in both normal mice and those with a severe combined immunodeficiency. Unfortunately, there are no more reports suggesting HDV infectivity in mice, probably as a consequence of the unproductive HBsAg binding to mNTCP receptor, as has been recently described.

#### 3.2.2. HDV Transgenic Mice

As previously described, HDV infection clearly increases the severity of liver pathology in patients with HBV chronic infection, but the molecular mechanism involved in the exacerbation of liver pathology associated with HDV infection is still unknown. Some authors suggest a potential cytophatic effect of this virus according to data obtained in chimpanzees, particularly during an acute infection [95]. Work performed by other groups suggested the potential direct pathogenic role of HDV antigens. S-HDAs have been shown to have a cytotoxic effect when overexpressed in HepG2 or HeLa cells, inducing the necrosis of the cells [96]. Additionally, it has been postulated that the l-HDAg might play a role in HDV pathogenesis due to its capacity to activate many eukaryotic promoters and modulate different signaling pathways [62,73,74]. In order to elucidate the role of HDV antigens in the exacerbation of liver disease, the group of Dr. Frank Chisari at Scripps, La Jolla, CA, developed transgenic mice for expression of S-HDAg or l-HDAg in the liver [97]. These animals express HDAgs in hepatocyte nuclei, as observed in natural HDV infection. However, no obvious signs of biological or histopathological evidence of liver disease, cytotoxicity, or hepatitis were detectable during the mice’s lifespan. Furthermore serum transaminase levels remained within the normal range, indicating that neither the large nor small form of HDAg are directly cytopathic to the hepatocyte* in vivo* [97]. Thus, contrary to expectations, HDAg* in vivo* does not seem have a pathological role.

Because HDV infection occurs mainly in the context of a coexisting HBV infection, the authors asked if the liver damage observed in patients with HDV infection might require the presence of both HDAgs and HBsAgs. To answer this question, the authors crossed HDAg transgenic mice with HBsAg transgenic mice. Once again no histopathological or biochemical evidence of liver cell injury was observed, indicating that viral proteins are not directly cytopathic to the hepatocyte. The authors suggest that maybe the presence of other HBV proteins is required to promote hepatocyte damage [97]. However, the role of the immune response against viral antigens should not be forgotten. Transgenic animals’ HDAg is expressed at fetal stages since the transgene is driven by an albumin promoter and the animals will recognize the viral antigen as a self-antigen, inducing a tolerogenic immune response. Moreover, liver injury associated with HDV infection could be a consequence of HDV and HBV protein and viral replication presence or the response of the host to the same factors.

After the transgenic animal model expressing HDAgs, a new transgenic mouse carrying a replication-competent HDV genomic dimer RNA was developed (HDVTg). HDVTg mice have been shown competent for studying HDV replication* in vivo* [98]. Surprisingly, despite the expression of HDV being under the control of the albumin promoter, HDV genomic and antigenomic sequences were detected in the liver, brain, testis, kidney, and particularly in skeletal muscle, where the amount of HDV RNA was 100-fold higher than in the liver. These results indicate that the transcriptional leakage associated with the albumin promoter allows HDV priming expression and consequently licenses HDV replication in several tissues. The presence of antigenomic HDV monomer RNA is possible only after processing and replication of the primary genomic dimer transcript; thus, the data in this transgenic animal model indicated that HDV can replicate in nearly all cell types. Interestingly, it is observed that HDV replicates more efficiently in muscle cells than in the hepatocytes, indicating that the hepatic specificity of HDV is due to the HBV-derived envelope. Moreover, analysis of HDV antigen expression showed a similar pattern, *i.e.*, HDV small antigen was detected mainly in the muscles, and in lower amounts in the liver. However, no large HDV antigen was detected in any tissue, indicating that no efficient HDV RNA editing occurs in these animals. Finally, and in accordance with the results observed in HDAg transgenic animals, no evidence of pathology was seen in muscle, brain, or liver tissue without any manifestation of necrosis or inflammation. These data indicate that HDV RNA replication and delta small antigen expression did not appear to cause any evident pathology and consequently HBVpresence might be required to induce hepatocyte damage. Unfortunately, there is no information about the potential immune response against viral antigens in transgenic animals that could help to understand the absence of liver damage and could determine if these animals could have any potential for analyzing HDV immune responses.

Although the NTCP gene is highly conserved between species, mouse NTCP does not contain molecular determinants required for viral entry [33]. Recent studies have shown that exogenous hNTCP expression in a mouse hepatoma cell line allows them to support HDV infection, but not HBV infection. Remarkably,* in vitro* HDV infection was also achieved in cell lines originated from other species and tissues expressing exogenous hNTCP, confirming that HDV human hepatotropism is restricted by the presence of the hNTCP receptor [32,33]. Therefore, generation of transgenic animals expressing hNTCP or a chimeric mNTCP replaced with hNTCP 84 to 87 residues in mouse hepatocytes would be potentially susceptible to natural HDV infection. However, these animals would not be susceptible to HBV infection as mouse hepatocytes have been shown refractory to HBV infection and they will not support the complete HDV life cycle.

#### 3.2.3. Mice with “Humanized” Livers

Another approach to study HDV virus replication requires the use of immunodeficient mice with human hepatocytes engrafted in their livers [20,55]. To generate mice with humanized livers, the investigators induced murine hepatocyte damage to create the space and the environment for engraftment and proliferation of human transplanted cells. The first model is based on the use of immunodeficient mice transgenic for the urokinase plasminogen activator (uPA-SCID), and the second model is based on Rag2-deficient mice and γ chain of interleukin 2 receptor (Il2rg) and lacking the the fumaryl acetoacetate hydrolase (Fah) gene. In both cases the animals suffer dramatic hepatocellular damage (death of a high percentage of mouse hepatocytes) and mice are rescued by transplantation of human hepatocytes [99]. The human cells residing in the mouse liver can proliferate and repopulate host liver tissue and they are susceptible to the establishment and maintenance of intrahepatic HDV mono-infection, increasing intrahepatic amounts of large HDAg; edited HDV RNA forms increased over time. Moreover, HBV superinfection of chimeric mice supporting HDV infection leads to the production of infective HDV virion in mice sera and an increase of HDAg-positive human hepatocytes, demonstrating intrahepatic HDV spreading [55]. Additionally, HDV infection reduces significantly the increase of HBV viremia and intrahepatic cccDNA loads in comparison with HBV mono-infected mice, as has been observed in patients. These mice have been used to show the ability of the NTCP inhibitor Myrcludex B to block HBV and HDV entry [20]. However, humanized mouse livers present several limitations:

1. The availability of primary human hepatocytes is limited and they cannot be propagated* in vitro*, which limits the number of available animals.
2. The susceptibility of primary human hepatocytes to HBV infection is highly variable and generally low, since these cells rapidly dedifferentiate, losing the expression of the NTCP receptor.
3. The engraftment of human hepatocytes in the liver of mice requires the use of immunodeficient mice, thus the interaction of the immune system and the virus is lost and consequently experiments are restricted to studies analyzing virus host–cell interactions.

#### 3.2.4. Development of Gene Delivery Vectors to Deliver HDV Genomes into Mice

*In vivo* gene delivery has been widely used for elucidating gene function and for creating disease animal models. It is usually performed by a non-viral or viral approach. The former approach mainly depends on the use of recombinant DNA or RNA, whereas the latter approach depends on the use of viral vectors. The nonviral delivery has several advantages such as less toxicity, less immunogenicity, and safer and easier to prepare. However, this approach has limited gene delivery efficiency and results in a short duration of transgene expression.

It has been observed that cloned plant viroids’ cDNA copies are infectious when they are introduced in host cells [100]. Similarly, although HDV replication proceeds without DNA intermediates, multimeric forms of HDV cDNA transcribed or introduced in the cells are able to initiate the normal HDV replication pathway [101,102]. Accordingly, when an HBV-infected chimpanzee was inoculated by direct injection into the liver with a recombinant plasmid containing a full-length HDV genome and one expression vector allowing the translation of HDAg, it resulted in a productive infection with appearance of high levels of HDV RNA and long and short HDVAgs in the liver [103]. HDV RNA and HDAg were also detected in serum following a similar kinetic to the one observed in an acute infection, generating virions with mature HDV particles characteristics and a marked transient suppression of the synthesis of HBV DNA replicative intermediates during the active phase of HDV, as is observed in HDV superinfection in both humans and chimpanzees [104,105]. These results suggest that transduction of animals with HDV expression vectors can recapitulate most of the HDV viral cycle.

As previously described, HDV transgenic animals have shown that viral replication* in vivo* is not restricted to the liver and is highly efficient in muscle cells [98]. Consequently, when cDNA dimers of HDV were inoculated intramuscularly into mice, HDV genomic RNA increased to substantial levels by week 7 post-injection; also, antigenomic sense HDV RNA and hepatitis delta antigen were present in myocytes’ nuclei, confirming HDV ubiquitous replication competence, as has been observed in transgenic animals. Interestingly, sera from DNA injected mice contained antibodies specific for HDAg, indicating the induction of an immunological response to the intracellularly expressed antigen, similar to the one observed when mice are inoculated with an HDAg expression vector [106]. When HDV cDNA expression vector is hydrodynamically injected in the liver (a technique that allows the transduction of 10–30% of hepatocytes) there is an increase of genomic and antigenomic HDV RNA for two weeks, indicating the existence of HDV RNA replication [107]. Thereafter there is a decrease in HDV genomic RNA from day 15 to 30, in clear contrast to what is observed when the vector is inoculated intramuscularly. These differences can be caused by the use of different promoters for initial HDV RNA transcription or by the stronger HDV replication in muscle in comparison to hepatocytes, as has been previously observed in transgenic mice. However, we cannot exclude the possibility that this can also be due to the differences in the mouse strain used in each experiment. A similar situation was previously described for HBV replication: C57BL/6 mice develop robust HBV replication after hydrodynamic injection of HBV plasmids, whereas HBV replication is transient in BALB/c mice [108]. Interestingly, shortly after HDV hydrodynamic injection, small HDAg is highly expressed and is detected in nuclei and cytoplasm. S-HDAg expression becomes undetectable 15 days after DNA inoculation, indicating that HDAg decline precedes HDV RNA reduction. In this animal model, posttranscriptional RNA editing occurs, leading to the appearance of the large form of the large delta of HDV. However, l-HDAg protein represents just 3% of the accumulated delta protein in the hepatocytes, lower than the levels detected in transfected cells and indicating a deficient editing that can promote a suboptimal replication of the HDV genome, which results in the disappearance of the HDV genome and antigens. Therefore, it should be clarified whether transient HDV replication observed in mouse livers after the HDV-replicative vector is hydrodynamically delivered is due to the mouse strain used for the experiments or to liver characteristics.

As we already indicate several times, the HDV virions require for their formation the hepatitis B virus surface antigen subunits provided by HBV co-infection or by the administration of an HBsAg-expressing vector. When HBV transgenic mice that produce HBV virions are used as hosts for hydrodynamic injection of HDV cDNA, HDV viral particles are detected in the serum [23]. In these mice, HDV replicates in hepatocytes and close to 30% of hepatocytes display a characteristic nuclear staining observed in HDV-infected cells. There is a good correlation between the level of intrahepatic replication and HDV virions’ RNA in the serum. However, as was observed in wild-type animals, HDV replication in hepatocytes that contain HBV-replicating virus do not promote an increase in ALT level, as is observed in humans and chimpanzees. Moreover, no anti-HDV antibodies were detected in the serum of these animals, which might be a consequence of the tolerogenic environment present in HBV transgenic mice livers. Very importantly, the majority of the HDV RNA was cleared by day 21 from the livers and serum. These animals were useful to demonstrate that pharmacologic prenylation inhibition can prevent the production of HDV virions* in vivo* [23].

Taking all these studies together, we can conclude that none of the murine models developed so far recapitulate all the features of chronic HDV infection.

In the last years several groups have developed HBV animal models using a recombinant virus carrying the HBV genome, like adenovirus (Ad) [109,110] or adenoassociated virus (AAV) [111,112], which acts as a shuttle vector for the delivery of the viral genome into the cells. In our laboratory we are currently working on the development of a similar strategy to create a model of HDV chronic infection; preliminary data indicates that by using this strategy, persistent HDV replication can be achieved in mice.

## 4. Conclusions

In conclusion, animal models are essential for the knowledge of HDV–host interactions as well as for the development of new therapeutic strategies (summarized in Table 1). Data obtained in cell culture are in clear contradiction with the data obtained in animal models. While overexpression of HDAgs in cells is associated with cytotoxicity, necrosis, and apoptosis, none of these effects are detected in transgenic animal models. In this review we showed that the animal models available nowadays that recapitulate HDV chronic infection are natural hosts susceptible to infection by members of the hepadnaviridae family that can be co-infected with human HDV. However, these animals present obvious ethical and experimental limitations. Murine models developed so far do not recapitulate all the characteristics of HDV infection, such as RNA editing, sustained viral load, or liver injury. Furthermore, several of them were developed in the context of immunosuppressed animals, precluding studies regarding the immune response against the virus. This review highlights the need for the development of new small animal models that better reflect the main feature of HDV chronic infection and that will allow a deeper analysis of HDV–host interaction as well as facilitate the development of new therapeutic agents and/or vaccines.

**Table 1:** Summary of the main characteristics of HDV animal models.

	Animal model	Main characteristics	References
Natural animal model	chimpanzees	Chimpanzees can be efficiently infected by HDV, coinfection and superinfection experiments have been performed resulting in moderately severe and severe liver damage, respectively.	79–81
	woodchucks	Several laboratories have shown that woodchucks chronically infected with WHV can be infected by HDV and produce new HDV virions using WHV surface antigen to form the envelope. This animal model recapitulate many of the characterists of HDV infection in humans.	76–78
	bats	HDV pseudotyped with surface proteins of bat hepadnavirus TBHBV is able to infect both primary hepatocytes, a pattern similar to the pattern observed with HDV-HBV	83
WT mice	wild-type & inmunodeficient mice	Mice injected with HDV virus obtained from woodchucks transiently infect and replicate in the liver	44
HDV-transgenic mice	S-HDAg transgenic mice	S-HDAg is expressed in hepatocytes and localized in the nucleus. No liver injury was observed, thus S-HDAg protein is not responsible for HDV inducing liver damage.	97
	L-HDAg transgenic mice	L-HDAg is expressed in hepatocytes an localized in the nucleus. No liver injury was observed, thus L-HDAg protein is not responsible for HDV inducing liver damage.	97
	HDV Ag x HBV sAg	The phenotype of these mice does not differ from the phenotype of the parents	97
	HDV genome under the control of the Albumin promoter	HDV replication and S-HDAg expression was detected in several organs including the liver, muscle, and brain. However, no large HDV large antigen were detected in any tissue, indicating that there was no efficient HDV RNA editing. Furthermore, no liver pathology was observed.	98
Humanized mouse models	Fah-/-RAG-/-IL-2Rg-/- + human hepatocytes	Establishment of HDV infection was highly efficient in both HBV-infected and naïve chimeric mice, representing an interesting animal model to study HDV-human hepatocyte interaction and new antivirals.	20,55,99
HDV-transfected mice	HDV plasmid hydrodynamic injection	In this animal model there is an increase of HDV genome and antigenome in the liver but only during the first two weeks after hydrodinamic injetion. There is no associated damage. Edition is observed in the liver.	107
HDV-transfected mice	HDV plasmid intramuscular injection	Sustained RNA accumulation at least for 7 weeks. HDAg antibody generation	106

## References

[1] Rizzetto M., Ciancio A. (2012). Epidemiology of hepatitis D. Semin. Liver Dis..

[2] Rizzetto M., Canese M.G., Arico S., Crivelli O., Trepo C., Bonino F., Verme G. (1977). Immunofluorescence detection of new antigen-antibody system (delta/anti-delta) associated to hepatitis B virus in liver and in serum of HBsAg carriers. Gut.

[3] Rizzetto M., Hoyer B., Canese M.G., Shih J.W., Purcell R.H., Gerin J.L. (1980). Delta agent: Association of delta antigen with hepatitis B surface antigen and RNA in serum of delta-infected chimpanzees. Proc. Natl. Acad. Sci. USA.

[4] Lee S.D., Wang J.Y., Wu J.C., Tsai Y.T., Lo K.J., Lai K.H., Tsay S.H., Govindarajan S. (1987). Hepatitis D virus (delta agent) superinfection in an endemic area of hepatitis B infection: Immunopathologic and serologic findings. Scand. J. Infect. Dis..

[5] Lin H.H., Liaw Y.F., Chen T.J., Chu C.M., Huang M.J. (1989). Natural course of patients with chronic type B hepatitis following acute hepatitis delta virus superinfection. Liver.

[6] Wedemeyer H. (2011). Hepatitis D revival. Liver Int..

[7] Rizzetto M., Alavian S.M. (2013). Hepatitis delta: The rediscovery. Clin. Liver Dis..

[8] Ciancio A., Rizzetto M. (2014). Chronic hepatitis D at a standstill: Where do we go from here?. Nat. Rev. Gastroenterol. Hepatol..

[9] Gaeta G.B., Stroffolini T., Smedile A., Niro G., Mele A. (2007). Hepatitis delta in Europe: Vanishing or refreshing?. Hepatology.

[10] Heidrich B., Deterding K., Tillmann H.L., Raupach R., Manns M.P., Wedemeyer H. (2009). Virological and clinical characteristics of delta hepatitis in central Europe. J. Viral Hepat..

[11] Servant-Delmas A., Le Gal F., Gallian P., Gordien E., Laperche S. (2014). Increasing prevalence of HDV/HBV infection over 15 years in France. J. Clin. Virol..

[12] Abbas Z., Khan M.A., Salih M., Jafri W. (2011). Interferon alpha for chronic hepatitis D. Cochrane Database Syst. Rev..

[13] Abbas Z., Memon M.S., Mithani H., Jafri W., Hamid S. (2014). Treatment of chronic hepatitis D patients with pegylated interferon: A real-world experience. Antivir. Ther..

[14] Alvarado-Mora M.V., Locarnini S., Rizzetto M., Pinho J.R. (2013). An update on HDV: Virology, pathogenesis and treatment. Antivir. Ther..

[15] Chang J., Taylor J.M. (2003). Susceptibility of human hepatitis delta virus RNAs to small interfering RNA action. J. Virol..

[16] Singh S., Gupta S.K., Nischal A., Khattri S., Nath R., Pant K.K., Seth P.K. (2012). Design of potential siRNA molecules for hepatitis delta virus gene silencing. Bioinformation.

[17] Abou-Jaoude G., Molina S., Maurel P., Sureau C. (2007). Myristoylation signal transfer from the large to the middle or the small HBV envelope protein leads to a loss of HDV particles infectivity. Virology.

[18] Abou-Jaoude G., Sureau C. (2007). Entry of hepatitis delta virus requires the conserved cysteine residues of the hepatitis B virus envelope protein antigenic loop and is blocked by inhibitors of thiol-disulfide exchange. J. Virol..

[19] Blanchet M., Sureau C., Labonte P. (2014). Use of FDA approved therapeutics with hNTCP metabolic inhibitory properties to impair the HDV lifecycle. Antivir. Res..

[20] Lütgehetmann M., Mancke L.V., Volz T., Helbig M., Allweiss L., Bornscheuer T., Pollok J.M., Lohse A.W., Petersen J., Urban S. (2012). Humanized chimeric uPA mouse model for the study of hepatitis B and D virus interactions and preclinical drug evaluation. Hepatology.

[21] Urban S., Bartenschlager R., Kubitz R., Zoulim F. (2014). Strategies to inhibit entry of HBV and HDV into hepatocytes. Gastroenterology.

[22] Bordier B.B., Marion P.L., Ohashi K., Kay M.A., Greenberg H.B., Casey J.L., Glenn J.S. (2002). A prenylation inhibitor prevents production of infectious hepatitis delta virus particles. J. Virol..

[23] Bordier B.B., Ohkanda J., Liu P., Lee S.Y., Salazar F.H., Marion P.L., Ohashi K., Meuse L., Kay M.A., Cas J.L. (2003). *In vivo* antiviral efficacy of prenylation inhibitors against hepatitis delta virus. J. Clin. Investig..

[24] Taylor J.M. (2006). Hepatitis delta virus. Virology.

[25] Kos A., Dijkema R., Arnberg A.C., van der Meide P.H., Schellekens H. (1986). The hepatitis delta (delta) virus possesses a circular RNA. Nature.

[26] Taylor J.M. (2006). Structure and replication of hepatitis delta virus RNA. Curr. Top. Microbiol. Immunol..

[27] Makino S., Chang M.F., Shieh C.K., Kamahora T., Vannier D.M., Govindarajan S., Lai M.M. (1987). Molecular cloning and sequencing of a human hepatitis delta (delta) virus RNA. Nature.

[28] Flores R., Grubb D., Elleuch A., Nohales M.Á., Delgado S., Gago S. (2011). Rolling-circle replication of viroids, viroid-like satellite RNAs and hepatitis delta virus: Variations on a theme. RNA Biol..

[29] Flores R., Ruiz-Ruiz S., Serra P. (2012). Viroids and hepatitis delta virus. Semin. Liver Dis..

[30] Ke A., Zhou K., Ding F., Cate J.H., Doudna J.A. (2004). A conformational switch controls hepatitis delta virus ribozyme catalysis. Nature.

[31] Deny P. (2006). Hepatitis delta virus genetic variability: From genotypes I, II, III to eight major clades?. Curr. Top. Microbiol. Immunol..

[32] Ni Y., Lempp F.A., Mehrle S., Nkongolo S., Kaufman C., Fälth M., Stindt J., Königer C., Nassal M., Kubitz R. (2014). Hepatitis B and D viruses exploit sodium taurocholate co-transporting polypeptide for species-specific entry into hepatocytes. Gastroenterology.

[33] Yan H., Peng B., He W., Zhong G., Qi Y., Ren B., Gao Z., Jing Z., Song M. (2013). Molecular determinants of hepatitis B and D virus entry restriction in mouse sodium taurocholate cotransporting polypeptide. J. Virol..

[34] Yan H., Zhong G., Xu G., He W., Jing Z., Gao Z., Huang Y., Qi Y., Peng B., Wang H. (2012). Sodium taurocholate cotransporting polypeptide is a functional receptor for human hepatitis B and D virus. Elife.

[35] Pellicoro A., Faber K.N. (2007). Review article: The function and regulation of proteins involved in bile salt biosynthesis and transport. Aliment. Pharmacol. Ther..

[36] Xia Y.P., Yeh C.T., Ou J.H., Lai M.M. (1992). Characterization of nuclear targeting signal of hepatitis delta antigen: Nuclear transport as a protein complex. J. Virol..

[37] Chang J., Nie X., Chang H.E., Han Z., Taylor J. (2008). Transcription of hepatitis delta virus RNA by RNA polymerase II. J. Virol..

[38] Tseng C.H., Lai M.M. (2009). Hepatitis delta virus RNA replication. Viruses.

[39] Taylor J.M. (2009). Replication of the hepatitis delta virus RNA genome. Adv. Virus Res..

[40] Lehmann E., Brueckner F., Cramer P. (2007). Molecular basis of RNA-dependent RNA polymerase II activity. Nature.

[41] Greco-Stewart V.S., Schissel E., Pelchat M. (2009). The hepatitis delta virus RNA genome interacts with the human RNA polymerases I and III. Virology.

[42] Cao D., Haussecker D., Huang Y., Kay M.A. (2009). Combined proteomic-RNAi screen for host factors involved in human hepatitis delta virus replication. RNA.

[43] MacNaughton T.B., Gowans E.J., McNamara S.P., Burrell C.J. (1991). Hepatitis delta antigen is necessary for access of hepatitis delta virus RNA to the cell transcriptional machinery but is not part of the transcriptional complex. Virology.

[44] Netter H.J., Kajino K., Taylor J.M. (1993). Experimental transmission of human hepatitis delta virus to the laboratory mouse. J. Virol..

[45] Lai M.M. (1995). The molecular biology of hepatitis delta virus. Annu. Rev. Biochem..

[46] Reid C.E., Lazinski D.W. (2000). A host-specific function is required for ligation of a wide variety of ribozyme-processed RNAs. Proc. Natl. Acad. Sci. USA.

[47] Weiner A.J., Choo Q.L., Wang K.S., Govindarajan S., Redeker A.G., Gerin J.L., Houghton M. (1988). A single antigenomic open reading frame of the hepatitis delta virus encodes the epitope(s) of both hepatitis delta antigen polypeptides p24 delta and p27 delta. J. Virol..

[48] Chen R., Linnstaedt S.D., Casey J.L. (2010). RNA editing and its control in hepatitis delta virus replication. Viruses.

[49] Hartwig D., Schütte C., Warnecke J., Dorn I., Hennig H., Kirchner H., Schlenke P. (2006). The large form of ADAR 1 is responsible for enhanced hepatitis delta virus RNA editing in interferon-alpha-stimulated host cells. J. Viral Hepat..

[50] Polson A.G., Bass B.L., Casey J.L. (1996). RNA editing of hepatitis delta virus antigenome by dsRNA-adenosine deaminase. Nature.

[51] Wong S.K., Lazinski D.W. (2002). Replicating hepatitis delta virus RNA is edited in the nucleus by the small form of ADAR1. Proc. Natl. Acad. Sci. USA.

[52] Macnaughton T.B., Lai M.M. (2002). Genomic but not antigenomic hepatitis delta virus RNA is preferentially exported from the nucleus immediately after synthesis and processing. J. Virol..

[53] Wang C.J., Chen P.J., Wu J.C., Patel D., Chen D.S. (1991). Small-form hepatitis B surface antigen is sufficient to help in the assembly of hepatitis delta virus-like particles. J. Virol..

[54] Freitas N., Salisse J., Cunha C., Toshkov I., Menne S., Gudima S.O. (2012). Hepatitis delta virus infects the cells of hepadnavirus-induced hepatocellular carcinoma in woodchucks. Hepatology.

[55] Giersch K., Helbig M., Volz T., Allweiss L., Mancke L.V., Lohse A.W., Polywka S., Pollok J.M., Petersen J., Taylor J. (2014). Persistent hepatitis D virus mono-infection in humanized mice is efficiently converted by hepatitis B virus to a productive co-infection. J. Hepatol..

[56] Davies S.E., Lau J.Y., O’Grady J.G., Portmann B.C., Alexander G.J., Williams R. (1992). Evidence that hepatitis D virus needs hepatitis B virus to cause hepatocellular damage. Am. J. Clin. Pathol..

[57] Sheldon J., Ramos B., Toro C., Rios P., Martinez-Alarcon J., Bottecchia M., Romero M., Garcia-Samaniego J., Soriano V. (2008). Does treatment of hepatitis B virus (HBV) infection reduce hepatitis delta virus (HDV) replication in HIV-HBV-HDV-coinfected patients?. Antivir. Ther..

[58] Babiker Z.O., Hogan C., Ustianowski A., Wilkins E. (2012). Does interferon-sparing tenofovir disoproxil fumarate-based therapy have a role in the management of severe acute hepatitis delta superinfection?. J. Med. Microbiol..

[59] Sureau C. (2010). The use of hepatocytes to investigate HDV infection: The HDV/HepaRG model. Methods Mol. Biol..

[60] Brazas R., Ganem D. (1996). A cellular homolog of hepatitis delta antigen: Implications for viral replication and evolution. Science.

[61] Wang Y.H., Chang S.C., Huang C., Li Y.P., Lee C.H., Chang M.F. (2005). Novel nuclear export signal-interacting protein, NESI, critical for the assembly of hepatitis delta virus. J. Virol..

[62] Mota S., Mendes M., Penque D., Coelho A.V., Cunha C. (2008). Changes in the proteome of Huh7 cells induced by transient expression of hepatitis D virus RNA and antigens. J. Proteomics.

[63] Mota S., Mendes M., Freitas N., Penque D., Coelho A.V., Cunha C. (2009). Proteome analysis of a human liver carcinoma cell line stably expressing hepatitis delta virus ribonucleoproteins. J. Proteomics.

[64] Greco-Stewart V.S., Thibault C.S., Pelchat M. (2006). Binding of the polypyrimidine tract-binding protein-associated splicing factor (PSF) to the hepatitis delta virus RNA. Virology.

[65] Sikora D., Greco-Stewart V.S., Miron P., Pelchat M. (2009). The hepatitis delta virus RNA genome interacts with eEF1A1, p54(nrb), hnRNP-L, GAPDH and ASF/SF2. Virology.

[66] Williams V., Brichler S., Khan E., Chami M., Dény P., Kremsdorf D., Gordien E. (2012). Large hepatitis delta antigen activates STAT-3 and NF-kappaB via oxidative stress. J. Viral Hepat..

[67] Choi S.H., Jeong S.H., Hwang S.B. (2007). Large hepatitis delta antigen modulates transforming growth factor-beta signaling cascades: Implication of hepatitis delta virus-induced liver fibrosis. Gastroenterology.

[68] Pugnale P., Pazienza V., Guilloux K., Negro F. (2009). Hepatitis delta virus inhibits alpha interferon signaling. Hepatology.

[69] Goto T., Kato N., Yoshida H., Otsuka M., Moriyama M., Shiratori Y., Koike K., Matsumura M., Omata M. (2003). Synergistic activation of the serum response element-dependent pathway by hepatitis B virus x protein and large-isoform hepatitis delta antigen. J. Infect. Dis..

[70] Lo K., Sheu G.T., Lai M.M. (1998). Inhibition of Cellular RNA polymerase II transcription by delta antigen of hepatitis delta virus. Virology.

[71] Yamaguchi Y., Filipovska J., Yano K., Furuya A., Inukai N., Narita T., Wada T., Sugimoto S., Konarska M.M., Handa H. (2001). Stimulation of RNA polymerase II elongation by hepatitis delta antigen. Science.

[72] Liao F.T., Lee Y.J., Ko J.L., Tsai C.C., Tseng C.J., Sheu G.T. (2009). Hepatitis delta virus epigenetically enhances clusterin expression via histone acetylation in human hepatocellular carcinoma cells. J. Gen. Virol..

[73] Mendes M., Perez-Hernandez D., Vazquez J., Coelho A.V., Cunha C. (2013). Proteomic changes in HEK-293 cells induced by hepatitis delta virus replication. J. Proteomics.

[74] Wang D., Pearlberg J., Liu Y.T., Ganem D. (2001). Deleterious effects of hepatitis delta virus replication on host cell proliferation. J. Virol..

[75] Heinicke L.A., Bevilacqua P.C. (2012). Activation of PKR by RNA misfolding: HDV ribozyme dimers activate PKR. RNA.

[76] Negro F., Korba B.E., Forzani B., Baroudy B.M., Brown T.L., Gerin J.L., Ponzetto A. (1989). Hepatitis delta virus (HDV) and woodchuck hepatitis virus (WHV) nucleic acids in tissues of HDV-infected chronic WHV carrier woodchucks. J. Virol..

[77] Casey J.L., Gerin J.L. (2006). The woodchuck model of HDV infection. Curr. Top. Microbiol. Immunol..

[78] Caselmann W.H. (1994). HBV and HDV replication in experimental models: Effect of interferon. Antivir. Res..

[79] Kos T., Molijn A., van Doorn L.J., van Belkum A., Dubbeld M., Schellekens H. (1991). Hepatitis delta virus cDNA sequence from an acutely HBV-infected chimpanzee: Sequence conservation in experimental animals. J. Med. Virol..

[80] Negro F., Bergmann K.F., Baroudy B.M., Satterfield W.C., Popper H., Purcell R.H., Gerin J.L. (1988). Chronic hepatitis D virus (HDV) infection in hepatitis B virus carrier chimpanzees experimentally superinfected with HDV. J. Infect. Dis..

[81] Gerin J.L. (2001). Animal models of hepatitis delta virus infection and disease. ILAR J..

[82] Engelke M., Mills K., Seitz S., Simon P., Gripon P., Schnölzer M., Urban S. (2006). Characterization of a hepatitis B and hepatitis delta virus receptor binding site. Hepatology.

[83] Drexler J.F., Geipel A., König A., Corman V.M., van Riel D., Leijten L.M., Bremer C.M., Rasche A., Cottontail V.M., Maganga G.D. (2013). Bats carry pathogenic hepadnaviruses antigenically related to hepatitis B virus and capable of infecting human hepatocytes. Proc. Natl. Acad. Sci. USA.

[84] Zhong G., Yan H., Wang H., He W., Jing Z., Qi Y., Fu L., Gao Z., Huang Y., Xu G. (2013). Sodium taurocholate cotransporting polypeptide mediates woolly monkey hepatitis B virus infection of Tupaia hepatocytes. J. Virol..

[85] Walter E., Keist R., Niederost B., Pult I., Blum H.E. (1996). Hepatitis B virus infection of tupaia hepatocytes *in vitro* and *in vivo*. Hepatology.

[86] Li Q., Ding M., Wang H. (1995). The infection of hepatitis D virus in adult tupaia. Zhonghua Yi Xue Za Zhi.

[87] Ponzetto A., Hoyer B.H., Popper H., Engle R., Purcell R.H., Gerin J.L. (1987). Titration of the infectivity of hepatitis D virus in chimpanzees. J. Infect. Dis..

[88] Chen P.J., Yang P.M., Chen C.R., Chen D.S. (1989). Characterization of the transcripts of hepatitis D and B viruses in infected human livers. J. Infect. Dis..

[89] Karayiannis P., Goldin R., Luther S., Carman W.F., Monjardino J., Thomas H.C. (1992). Effect of cyclosporin-A in woodchucks with chronic hepatitis delta virus infection. J. Med. Virol..

[90] Karayiannis P., Saldanha J., Monjardino J., Jackson A., Luther S., Thomas H.C. (1993). Immunisation of woodchucks with hepatitis delta antigen expressed by recombinant vaccinia and baculoviruses, controls HDV superinfection. Prog. Clin. Biol. Res..

[91] Fiedler M., Lu M., Siegel F., Whipple J., Roggendorf M. (2001). Immunization of woodchucks (Marmota monax) with hepatitis delta virus DNA vaccine. Vaccine.

[92] Fiedler M., Roggendorf M. (2001). Vaccination against hepatitis delta virus infection: Studies in the woodchuck (Marmota monax) model. Intervirology.

[93] Fiedler M., Kosinska A., Schumann A., Brovko O., Walker A., Lu M., Johrden L., Mayer A., Wildner O., Roggendorf M. (2013). Prime/boost immunization with DNA and adenoviral vectors protects from hepatitis D virus (HDV) infection after simultaneous infection with HDV and woodchuck hepatitis virus. J. Virol..

[94] D’Ugo E., Paroli M., Palmieri G., Giuseppetti R., Argentini C., Tritarelli E., Bruni R., Barnaba V., Houghton M., Rapicetta M. (2004). Immunization of woodchucks with adjuvanted sHDAg (p24): Immune response and outcome following challenge. Vaccine.

[95] Govindarajan S., Fields H.A., Humphrey C.D., Margolis H.S. (1986). Pathologic and ultrastructural changes of acute and chronic delta hepatitis in an experimentally infected chimpanzee. Am. J. Pathol..

[96] Cole S.M., Macnaughton T.B., Gowans E.J. (1993). Differential roles for HDAg-p24 and -p27 in HDV pathogenesis. Prog. Clin. Biol. Res..

[97] Guilhot S., Huang S.N., Xia Y.P., La Monica N., Lai M.M., Chisari F.V. (1994). Expression of the hepatitis delta virus large and small antigens in transgenic mice. J. Virol..

[98] Polo J.M., Jeng K.S., Lim B., Govindarajan S., Hofman F., Sangiorgi F., Lai M.M. (1995). Transgenic mice support replication of hepatitis delta virus RNA in multiple tissues, particularly in skeletal muscle. J. Virol..

[99] Grompe M., Strom S. (2013). Mice with human livers. Gastroenterology.

[100] Navarro B., Gisel A., Rodio M.E., Delgado S., Flores R., Di Serio F. (2012). Viroids: How to infect a host and cause disease without encoding proteins. Biochimie.

[101] Chen P.J., Kuo M.Y., Chen M.L., Tu S.J., Chiu M.N., Wu H.L., Hsu H.C., Chen D.S. (1990). Continuous expression and replication of the hepatitis delta virus genome in Hep G2 hepatoblastoma cells transfected with cloned viral DNA. Proc. Natl. Acad. Sci. USA.

[102] Kuo M.Y., Chao M., Taylor J. (1989). Initiation of replication of the human hepatitis delta virus genome from cloned DNA: Role of delta antigen. J. Virol..

[103] Sureau C., Taylor J., Chao M., Eichberg J.W., Lanford R.E. (1989). Cloned hepatitis delta virus cDNA is infectious in the chimpanzee. J. Virol..

[104] Wu J.C., Chen T.Z., Huang Y.S., Yen F.S., Ting L.T., Sheng W.Y., Tsay S.H., Lee S.D. (1995). Natural history of hepatitis D viral superinfection: Significance of viremia detected by polymerase chain reaction. Gastroenterology.

[105] Dienes H.P., Purcell R.H., Popper H., Ponzetto A. (1990). The significance of infections with two types of viral hepatitis demonstrated by histologic features in chimpanzees. J. Hepatol..

[106] Polo J.M., Lim B., Govindarajan S., Lai M.M. (1995). Replication of hepatitis delta virus RNA in mice after intramuscular injection of plasmid DNA. J. Virol..

[107] Chang J., Sigal L.J., Lerro A., Taylor J. (2001). Replication of the human hepatitis delta virus genome is initiated in mouse hepatocytes following intravenous injection of naked DNA or RNA sequences. J. Virol..

[108] Lin Y.J., Huang L.R., Yang H.C., Tzeng H.T., Hsu P.N., Wu H.L., Chen P.J., Chen D.S. (2010). Hepatitis B virus core antigen determines viral persistence in a C57BL/6 mouse model. Proc. Natl. Acad. Sci. USA.

[109] Huang L.R., Gäbel Y.A., Graf S., Arzberger S., Kurts C., Heikenwalder M., Knolle P.A., Protzer U. (2012). Transfer of HBV genomes using low doses of adenovirus vectors leads to persistent infection in immune competent mice. Gastroenterology.

[110] Xia Y., Zeng D., Yu F., He J., Zhou Z., Tu W., Deng H., Tian D.A., Liu M. (2012). Role of autophagy in monokine induced by interferon gamma (Mig) production during adenovirus-hepatitis B virus infection. Hepatogastroenterology.

[111] Dion S., Bourgine M., Godon O., Levillayer F., Michel M.L. (2013). Adeno-associated virus-mediated gene transfer leads to persistent hepatitis B virus replication in mice expressing HLA-A2 and HLA-DR1 molecules. J. Virol..

[112] Huang Y.H., Fang C.C., Tsuneyama K., Chou H.Y., Pan W.Y., Shih Y.M., Wu P.Y., Chen Y., Leung P.S., Gershwin M.E. (2011). A murine model of hepatitis B-associated hepatocellular carcinoma generated by adeno-associated virus-mediated gene delivery. Int. J. Oncol..

